# Depth-Resolved Variations of Cultivable Bacteria and Their Extracellular Enzymes in the Water Column of the New Britain Trench

**DOI:** 10.3389/fmicb.2018.00135

**Published:** 2018-02-06

**Authors:** Qianfeng Liu, Jiasong Fang, Jiangtao Li, Li Zhang, Bin-Bin Xie, Xiu-Lan Chen, Yu-Zhong Zhang

**Affiliations:** ^1^State Key Laboratory of Marine Geology, Tongji University, Shanghai, China; ^2^Hadal Science and Technology Research Center, Shanghai Ocean University, Shanghai, China; ^3^Laboratory for Marine Biology and Biotechnology, Qingdao National Laboratory for Marine Science and Technology, Qingdao, China; ^4^Department of Natural Sciences, Hawaii Pacific University, Honolulu, HI, United States; ^5^State Key Laboratory of Geological Process and Mineral Resources, Faculty of Earth Sciences, China University of Geosciences, Wuhan, China; ^6^State Key Laboratory of Microbial Technology, Marine Biotechnology Research Center, Shandong University, Jinan, China

**Keywords:** cultivable bacteria, extracellular enzymes, inhibitor analysis, *V*_max_, *K*_m_, aminopeptidase, New Britain Trench

## Abstract

Marine microorganisms and their extracellular enzymes (ECEs) play an important role in the remineralization of organic material by hydrolyzing high-molecular-weight substrates to sizes sufficiently small to be transported through cell membrane, yet the diversity of the enzyme-producing bacteria and the types of ECEs involved in the degradation process are largely unknown. In this work, we investigated the diversity of cultivable bacteria and their ECEs and the potential activities of aminopeptidase in the water column at eight different depths of the New Britain Trench. There was a great diversity of cultivable bacteria and ECEs, and depth appears an important driver of the diversity. The 16S rRNA sequence analysis revealed that the cultivable bacteria were affiliated mostly with the phyla Proteobacteria and Actinobacteria, and the predominant genera were *Pseudoalteromonas* (62.7%) and *Halomonas* (17.3%). Moreover, 70.7% of the isolates were found to produce hydrolytic zone on casein and gelatin plates, in which *Pseudoalteromonas* was the predominant group, exhibiting relatively high protease production. Inhibitor analysis showed that the extracellular proteases from the isolated bacteria were serine proteases in the surface water and metalloproteases in the deep water. Meanwhile, the *V*_max_ and *K*_m_ of aminopeptidase exhibited a maximum in the surface water and low values in the deep bathy- and abyssopelagic water, indicating lower rates of hydrolysis and higher substrate affinity in the deeper waters. These results shed new insights into the diversity of the cultivable bacteria and bacterial ECEs and their likely biogeochemical functions in the trench environment.

## Introduction

Organic matter in the ocean's photic zone is mostly remineralized in the upper layers of the water column, and an estimated 0.1% of the production exported downward through the water column and ultimately buried in deep-sea sediments (Hedges, 1992). Heterotrophic microbial communities decompose particles and aggregates of marine organic matter and shape the nature and quantity of the released carbon and nutrients that pass through the water column from surface water to the deep ocean (Azam, 1998). Recent investigations on the role of marine bacteria in carbon cycling in the mesopelagic and bathypelagic waters have greatly contributed to our understanding of the importance of microbial ECEs in the biogeochemical processes of the global ocean (Zoppini et al., 2005; Ziervogel et al., 2007; Baltar et al., 2009; Steen et al., 2010; Arnosti, 2011, 2014; Williams et al., 2013).

Marine carbon cycle is driven largely by unicellular microorganisms including bacteria and archaea (Arístegui et al., 2009). Bacteria mediate a significant flux of organic matter, from particulate organic matter (POM) to dissolved organic matter (DOM), and account for a substantial fraction of heterotrophic respiration in the oceans (del Giorgio et al., 1997; Ducklow et al., 2009). About 50% of the organic carbon produced in the euphotic zone is processed by bacteria and used to produce new bacterial biomass and satisfy the energy requirements for bacterial respiration (Ducklow and Carlson, 1992). The POM-DOM Piezophilic Microorganism Continuum (PDPMC) model proposed by Fang et al. (2015) suggests that microorganisms in the deep ocean play a more important role in mineralization of marine organic matter than hitherto recognized (Nagata, 2000; Tamburini et al., 2003, 2009, 2013; Glud et al., 2013).

The sinking marine organic matter produced in the surface ocean is a composite of macromolecular compounds, including structural carbohydrates, proteins, nucleic acids, and lipid complexes (Jørgensen, 2000). Because only sufficiently small substrates (molecular weight <600 Da; Weiss and Abele, 1991) could be transported through microbial cell walls for further processing, microbial extracellular enzymes (ECEs) are implicated in catalyzing the breakdown of POC and transforming high-molecular-weight biopolymers to low-molecular-weight molecules. Thus, the activity of ECEs is considered to be the limiting factor for heterotrophic remineralization of organic matter (Chrost, 1991). Previous work has focused on measurements of enzyme activities (e.g., aminopeptidase, phosphatase, α- and β- glucosidase) of heterotrophic microbial community in shallow water (Davey et al., 2001; Tamburini et al., 2003; Steen and Arnosti, 2013; Li et al., 2015). There are few reports of enzyme activity of the deeper meso- and bathypelagic zones (Nagata et al., 2010; Zaccone et al., 2012; Baltar et al., 2013). Otherwise, more and more studies have been done to understand the diversity of cultivable species and their extracellular enzymes from marine sediments, of which most studies were focused on the diversity of protease-producing bacteria and their proteases in marine sediments (Olivera et al., 2007; Zhou et al., 2009; Li et al., 2017). In these studies, *Gammaproteobacteria* were the predominant cultivable protease-producing bacteria in sediments of the sub-Antatarctic (Olivera et al., 2007), the South China sea (Zhou et al., 2009) and the Laizhou Bay in China (Li et al., 2017). What is more, nearly all the extracellular proteases secreted by these cultivable protease-producing bacteria were serine and/or metalloproteases (Zhou et al., 2009, 2013; Zhang et al., 2015; Li et al., 2017). However, systematic investigations on the diversity of enzyme-producing bacteria and bacterial ECEs are lacking in the deep abyssal water column.

The trenches, with their unique tectonics, topography, bathymetry, hydrography, and microbiology, probably play an important role in the global ocean carbon cycle (Jamieson and Fujii, 2011; Ichino et al., 2015; Liu et al., 2017). The New Britain Trench (NBT), close to the landmass of Papua New Guinea, is an 840-km-long curved trench in the northern Solomon Sea. Recent research reveals that the New Britain Trench receives substantially more allochthonous input of organic matter from terrestrial sources than other trenches (e.g., the Mariana Trench) and correspondingly, is overlain by waters with higher net primary productivity and higher faunal abundance (Gallo et al., 2015). Thus, the New Britain Trench offers a rare opportunity to observe the variations of microbial community structure and enzymatic diversity in the water column. We chose to utilize this site, hypothesizing that the cultivable bacteria and their extracellular enzymes would exhibit stratified profiles in the water column. To test the hypothesis, we collected water samples from eight different depths and investigated the diversity and variations of cultivatable bacteria and their ECEs. To the best of our knowledge, this is the first report of cultivable enzyme-producing bacteria and their enzymatic activity in the abyssal water column of a Hadal Trench.

## Methods

### Sampling area and geochemical characteristics of samples

Water samples were taken from the New Britain Trench at station E (06°02.1243′S, 151°58.5042′E) in an August 2016 cruise aboard the M/V *Zhang Jian*. The sampling station is located in the northern Solomon Sea, close to the landmass of Papua New Guinea. Samples from eight different depths (75, 200, 1000, 2000, 3000, 4000, 5000, and 6000 m) were collected using Niskin bottles fitted on a Sea-Bird Carousel equipped with a conductivity-temperature-depth (CTD) sensor (Sea-Bird SBE 911). For microbiological analysis, about 1.5–2 L water sample from each depth was filtered through a 0.22 μm pore size polycarbonate (PC) membrane (47 mm, Millipore) to collect bacterial assemblages. The membranes were stored in sterile cryo-tubes at 4°C until further processing. POC and PON contents were obtained by filtering 7–9 L seawater through 25 mm glass fiber filters of 0.7 μm nominal pore size (GF-75, Whatman) which were pre-combusted in a muffle furnace at 500°C for 12 h. The membranes were stored at−20°C for laboratory analysis of POC and PON with a PE2400 Series II CHNS/O analyzer (Perkin Elmer, USA) (Chen et al., 2008).

### Bacterial counts and aminopeptidase activity measurements

Subsamples of seawater from each depth were immediately fixed in 4% (v/v) glutaraldehyde after retrieval of the samples and stored at −20°C on board. In laboratory, cells were filtered onto a 0.22 μm pore sized black polycarbonate filters (diameter, 25 mm; Whatman-Nucleopore) and stained with DAPI (4′,6-diamidino-2-phenylindole; Porter and Feig, 1980; Turley, 1993). Bacterial cells were counted under epifluorescent microscopy (Nikon microscope, model Eclipse Ni-U).

The fluorogenic substrate _L_-leucine-7-amino-4-methylcoumarin (Leu-MCA; Sigma) was used to measure aminopeptidase activity of seawater from the eight depths (Hoppe, 1983, 1993). Substrate was added in triplicate seawater samples to give final concentrations of 2.5, 5, 10, 50, and 100 μM. All samples were incubated in 4.5 ml cuvettes at *in situ* temperatures in the dark. Fluorescence was measured at 0, 3, and 6 h using a spectrofluorometer (Hitachi, model F-4500) at 380 nm excitation and 440 nm emission. A calibration curve was run using a series of standard solutions of 7-amino-4-methylcoumarin (MCA; Sigma) in seawater.

The maximum rate of hydrolysis (*V*_max_) and the half-saturation constant (*K*_m_) were calculated using non-linear regression (Lineweaver-Burk plot method, GraphPad Prism 5.01) based on Michaelis-Menten kinetics with triplicate sets of seawater samples. Differences in *V*_max_ and *K*_m_ between surface waters (75 and 200 m) and deep waters (1000–6000 m) were analyzed statistically using Mann–Whitney Test (non-parametric test; GraphPad PRISM version 5.01, Graphpad Software).

### Cultivation of bacteria from seawater samples

PC membrane of each depth was cut into strips (~2 × 10 mm) with autoclaved scissors and put into autoclaved conical flask containing micro glass beads and 15 ml saline solution (containing 2.75% NaCl, 0.5% MgCl_2_, 0.2% MgSO_4_, 0.05% CaCl_2_, 0.1% KCl, 0.0001% FeSO_4_, and distilled water) (Smibert and Krieg, 1994). After be incubated at 100 rpm and 15°C for 40 min, each conical flask was serially diluted 10-fold to 10^−6^ dilution with a sterile saline solution. Aliquots of 100 μl diluted deep sea water samples (10^−2^ to 10^−5^) were spread on enriched medium containing 0.5% trypton, 0.1% yeast extract, 1.5% agar powder and artificial seawater (pH 7.0). After being incubated at 15°C for 5–7 days, morphologically different colonies were selected and further purified by repeatedly streaking on the same medium. The purified strains were stored in 20% glycerol at −80°C for use.

### Amplification of 16s rRNA genes and phylogenetic analysis

Genomic DNA from bacterial isolates was obtained using a bacterial genomic DNA isolation kit (BioTeke). The 16S rRNA genes were amplified by polymerase chain reaction from genomic DNA with the universal primers 27F (59-AGAGTTTGATCCTGGCTCAG-39) and 1492R (59-ACGGCTACCTTGTTACGACTT-39) (Lane, 1991). These genes were ligated into pGEM-T cloning vectors (Promega) and sequenced by Sain Biological Corporation (Shanghai, China). Sequence alignment was performed using CLUSTAL X (v 1.83). Isolates with two or more different bases in their 16S rRNA gene sequences were taken as different strains. Neighbor-joining trees were constructed using MEGA5 (Kumar et al., 2008) with neighbor-joining method and Kimura two parameter model.

### Analysis of hydrolytic ability of enzymes to casein, gelatin, starch, and triacetin

Plates were prepared with four different media: basic medium (0.2% yeast extract, 1.5% agar powder, and artificial seawater, pH 8.0) with 0.5% (w/v) casein, 1% (w/v) gelatin, 1% (w/v) starch, and 2% (v/v) triacetin, respectively. Strains were streaked on the four different plates containing casein, gelatin, starch or triacetin, and incubated at 15°C for 4 days. Then, for each strain, the diameter of its colony (C) and the diameter of the hydrolytic zone (H) it produced were measured, and the ratio of the hydrolytic zone diameter to the colony diameter (hydrolytic zone/colony, H/C) was calculated (Zhou et al., 2013).

### Analysis of the inhibitory effect of protease inhibitors on protease activity

The protease-producing strains were incubated in the liquid medium (0.3% casein, 0.5% gelatin, 0.2% yeast extract and artificial sea water, pH 8.0) at 15°C 180 rpm for 3 days. After centrifuged at 12,000 × g, 4°C for 10 min, the protease activity of the supernatant was measured (Chen et al., 2003). One unit of enzyme activity was defined as the amount of enzyme that catalyzed the formation of 1 μg tyrosine per minute. The supernatant was pre-incubated with 1.0 mM phenylmethylsulfonyl fluoride (PMSF; Sigma) and 1.0 mM 1,10-phenanthroline (OP; Sigma) at 4°C for 45 min, respectively. After incubation, the protease activity of every sample was measured. The activity of a sample without any inhibitor was taken as 100%, and the relative activity (%) of samples was calculated. The inhibition ratio was taken as the result of control activity minus the relative activity of a sample (Zhou et al., 2009).

### Nucleotide sequence accession numbers

The 16S rRNA gene sequences resulting from this work were deposited in GenBank with the accession numbers KY744362-KY744436.

## Results

### Concentrations of POC and PON and bacterial enumeration

**Figure 1:**
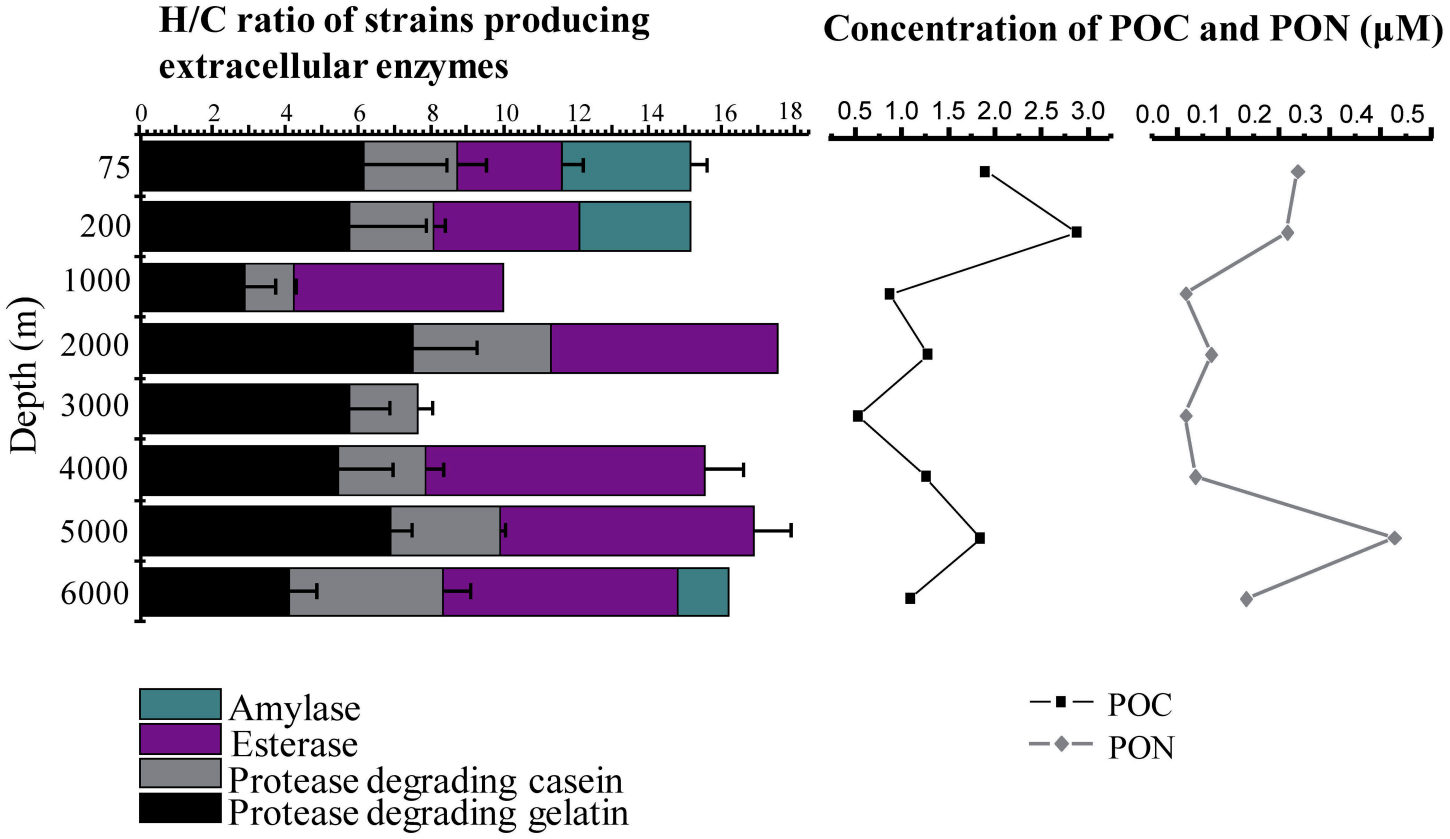
Depth profiles of mean H/C ratios of extracellular enzymes secreted by the cultivable bacteria and concentrations of POC and PON in the water column of the New Britain Trench.

The concentrations of POC and PON were in the range of 0.54–2.89 and 0.02–0.43 μM, respectively. The highest values were observed at the 200 and 5000 m depths, and the lowest values at the depths of 1000 and 3000 m (Figure 1). As expected, bacterial counts were higher in surface water (75 and 200 m), ranging from 1.23 × 10^5^ to 0.68 × 10^5^ cells ml^−1^, and low in the deep water (1000–6000 m), ranging from 4.5 × 10^3^ to 13.7 × 10^3^ cells ml^−1^ (Table 1, Figure S3).

**Table 1:** Depth profiles of the maximum potential proteolytic extracellular enzyme activity and activity per cell in the water column of the New Britain Trench.

**Depth (m)**	**Temperature (°C)**	**Proteolytic enzyme**	**Bacterial numbers**	**Cell-specific activity**
		***V*_max_ (pmol L^−1^ h^−1^)**	**(× 10^3^ cells ml^−1^ ± *SD*)**	**(pmol cell^−1^ h^−1^)**
75	26.5	4,136	123.7 ± 12.26	0.033
200	17.1	2,777	68.1 ± 16.06	0.041
1000	4.3	610	13.1 ± 1.15	0.047
2000	2.3	485	10.5 ± 0.82	0.046
3000	1.9	78	5.7 ± 0.37	0.014
4000	2.0	206	13.7 ± 1.59	0.015
5000	2.1	368	4.5 ± 0.15	0.082
6000	2.3	362	9.9 ± 0.47	0.037

### Diversity of cultivable bacteria in the water column of the new britain trench

After incubated on rich medium, colonies appeared on plates of the 10^−1^ to 10^−3^ diluted samples. Quantitative statistics by manual count showed that 10^2^ to 10^4^ bacterial cells ml^−1^ could be cultivated from the seawater of different depths. A total 202 colonies were selected and purified from all the plates. Nearly complete 16S rRNA genes of purified colonies were amplified and sequenced. Isolates with two or more different bases in their 16S rRNA gene sequences were considered as different strains, and finally sequence analysis identified 75 unique strains.

**Figure 2:**
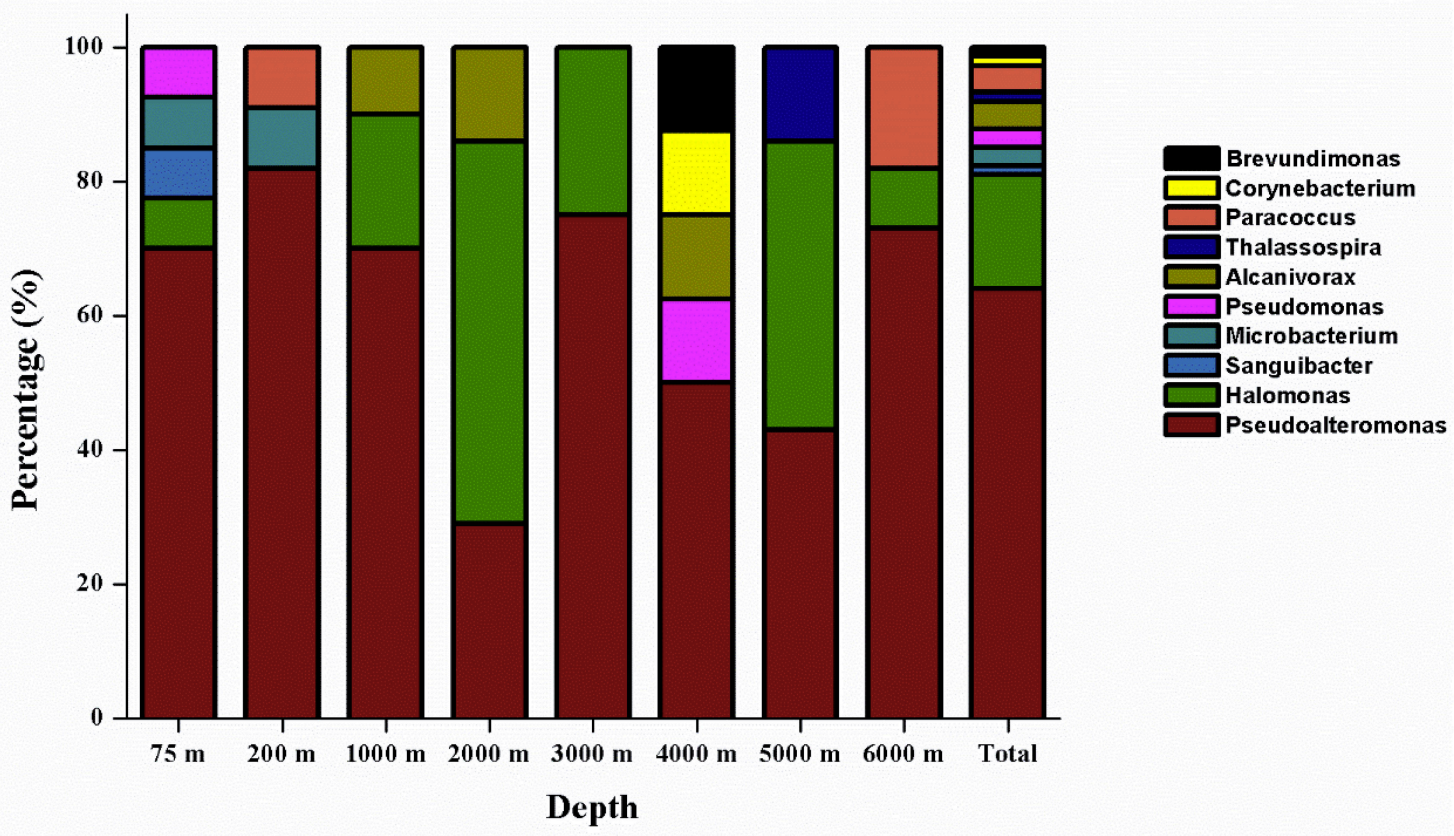
Relative percentage abundances of the phylotypic groups of cultivable bacteria recovered from seawater at eight different depths in the New Britain Trench.

**Figure 3:**
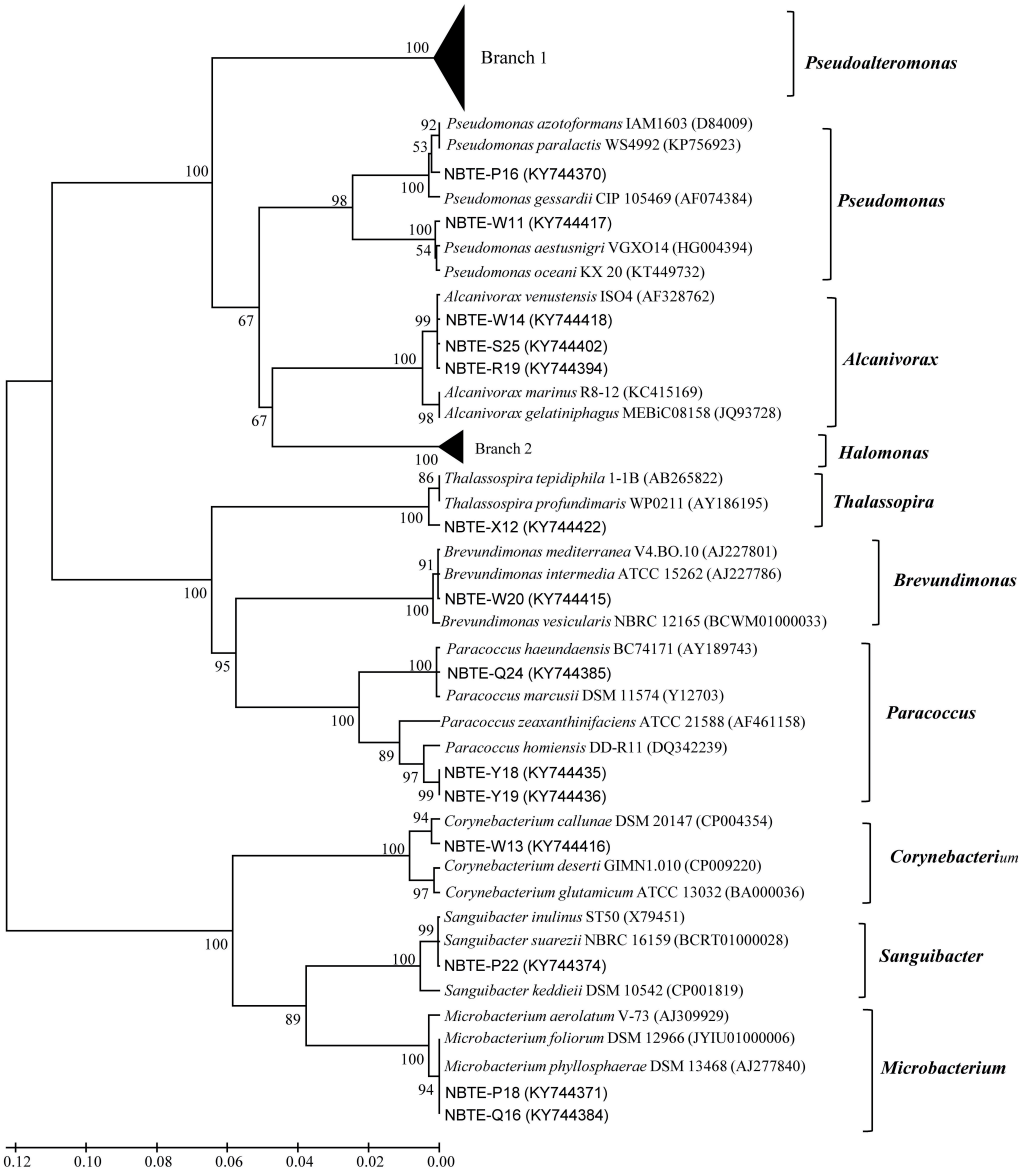
Neighbor-joining phylogenetic tree of cultivable bacterial strains from eight different depths in the New Britain Trench based on 16S rDNA sequences.

The phylogenetic affiliation of the 75 isolated strains was analyzed according to their 16S rRNA genes. Except the four gram-positive bacterial strains (NBTE-P18, NBTE-Q16, NBTE-P22, and NBTE-W13) belonging to phylum Actinobacteria, the rest of the isolates were affiliated with the phylum Proteobacteria and grouped in the genera *Pseudoalteromonas, Pseudomonas, Alcanivorax, Halomonas, Thalassospira, Brevundimonas*, and *Paracoccus*. *Pseudoalteromonas* (62.7%) and *Halomonas* (17.3%) were the predominant groups (Figure 2). In addition, *Pseudoalteromonas* dominated the cultivable fraction of the microbial communities in all eight depths except 2000 m where *Halomonas* was the predominant group (Figure 2). A distance-based neighbor-joining tree was constructed with sequences of the 75 cultivable strains and reference sequences from the GenBank database (Figure 3). Strains related to *Pseudoalteromonas* were the most frequently recovered isolates from the eight depths and formed the largest group in terms of abundance (47 of 75 isolates; Branch 1 in Figure 3, Figure S1). Thirteen *Halomonas* strains were closely related to *Halomonas merididiana* DSM 5245, *Halomonas aquamarina* DSM 30161 and *Halomonas axialensis* Althfl (Branch 2 in Figure 3, Figure S2). The phylogenetic relationship of other strains to their closely related species is also shown in Figure 3. Several strains, including NBTE-P16, NBTE-W11, and NBTE-X12, exhibited a distant relationship with the previously identified species, suggesting that these stains may represent potentially new species.

### Diversity of bacterial ECEs

**Figure 4:**
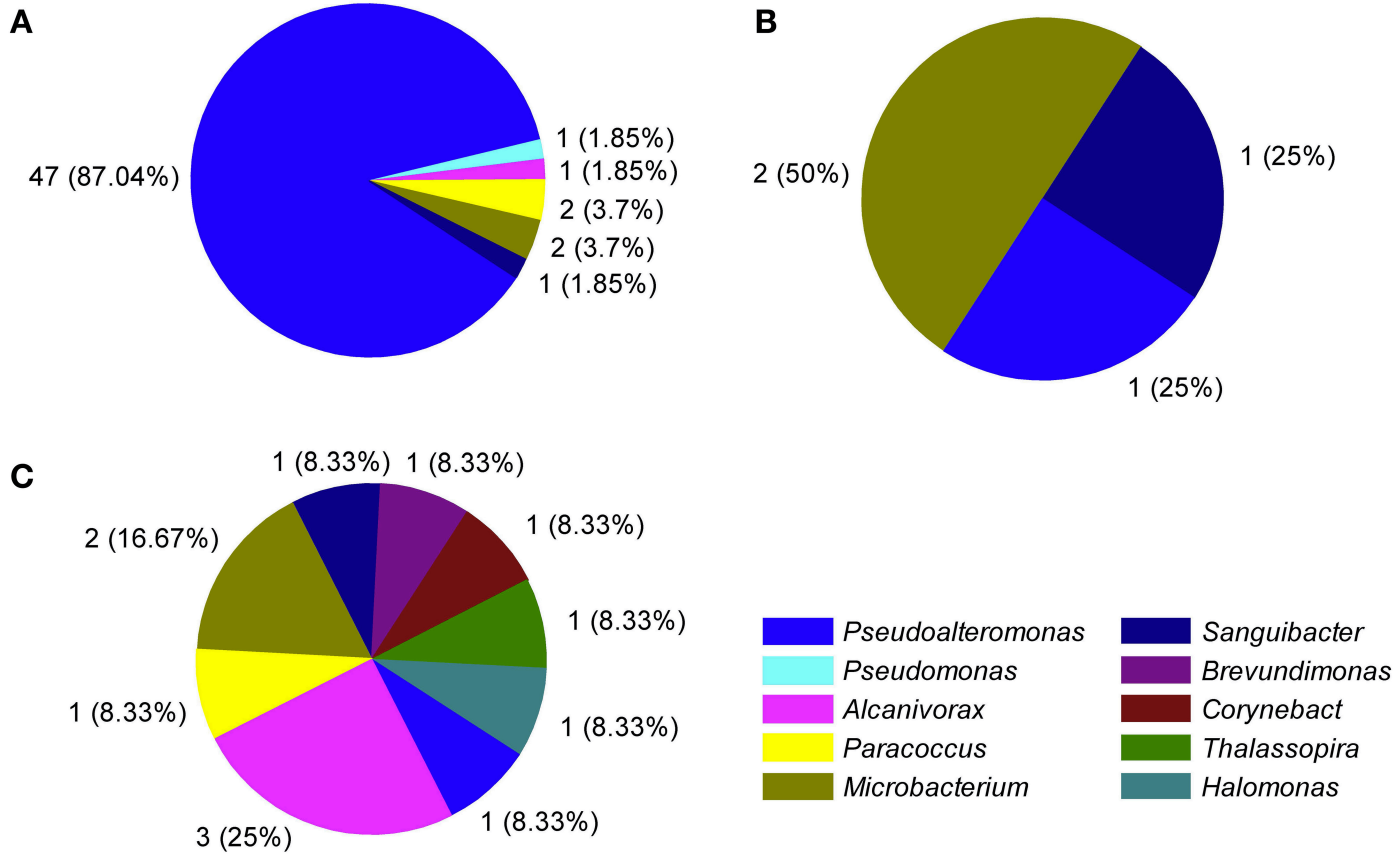
Distribution of the number (% abundant) of different cultivable genera producing each of the detected extracellular enzymatic activities. **(A)**, protease; **(B)**, amylase; **(C)**, esterase.

The diversity of the bacterial ECEs was investigated based on their ability of degrading the four different substrates, casein, gelatin, starch, or triacetin. Among the 75 bacterial isolates, we detected producers of casinse (44), gelatinolytic enzyme (53), amylase (4), and esterase (12). Additionally, although 53 strains could produce protease (degrading either casein or gelatin), these proteases displayed very different hydrolytic abilities to casein and gelatin. Forty-four of the 53 strains could degrade both casein and gelatin while the remaining nine strains could only degrade gelatin (Table S1). It appears that the cultivable enzyme-producing bacteria isolated from different depths exhibited different capacity of secreting enzymes. For example, the three strains, NBTE-P18, NBTE-P22, and NBTE-Q16 that degrade all three substrates (gelatin, starch, and triacetin) were from the surface water (75 and 200 m), whereas the eight strains degrading only triacetin were from the bathy- and abyssopelagic waters (2000, 4000, 5000, and 6000 m; Table S1). All four tested enzymes, amylase, esterase, protease degrading casein, and gelatin, were detected in the surface water (75 and 200 m) and the lower part of the abyssopelagic water (6000 m), and only three of the four enzymes were detected in the bathypelagic and the upper part of the abyssopelagic water (4000–5000 m) (Figure 1). The H/C ratio was used to evaluate the capabilities of the bacterial ECEs. Table S1 shows the protease diversity and activity of the bacteria isolates evaluated based on the H/C ratio against the four supplemented substrates, casein, gelatin, starch, and triacetin. Among the 53 protease-producing isolates, 26 strains exhibited high gelatinolytic activity with the H/C ratio > 5 (Li et al., 2017), and only 1 casein-degraders (NBTE-Y1) had high caseinolytic activity (with H/C ratio > 5). In general, the H/C ratios of triacetin-degraders from the bathypelagic waters were higher than that from the surface water (Figure 1). Among all the strains producing proteases, 86.8% belong to the genera *Pseudoalteromonas* (Figure 4) and all the isolated *Pseudoalteromonas* strains but NBTE-X19 and NBTE-T11 produced proteases. On the contrary, *Halomonas* strains could not produce any ECEs except NBTE-X22, which could degrade triacetin (Figure S2, Table S1).

To further investigate the diversity of bacterial extracellular protease secreted by the 53 protease-producing strains, PMSF (serine protease inhibitor) and OP (metalloprotease inhibitor) were used to inhibit the activities of the proteases. Among the 53 isolates, 13 strains could not produce enough extracellular protease for inhibition analysis, and the activities of the other 40 strains were inhibited by 38.0–99.8% with PMSF (Table S1). It is clear that the protease activity was inhibited by PMSF much less in abyssopelagic water (inhibition ratio of 38.0–48.6%) than the water above (inhibition ratio 77.1–99.8%, with an outlier of 25.5 at 4000 m). The opposite is true, i.e., inhibition of protease activity by OP was more in the abyssopelagic water than the shallow water, even though the inhibition ratio varied much more than does the PMSF inhibition ratio in all waters (Table S1). Thus, nearly all the extracellular proteases of the bacterial isolates belonged to either serine proteases or metalloproteases.

### Aminopeptidase activity

**Figure 5:**
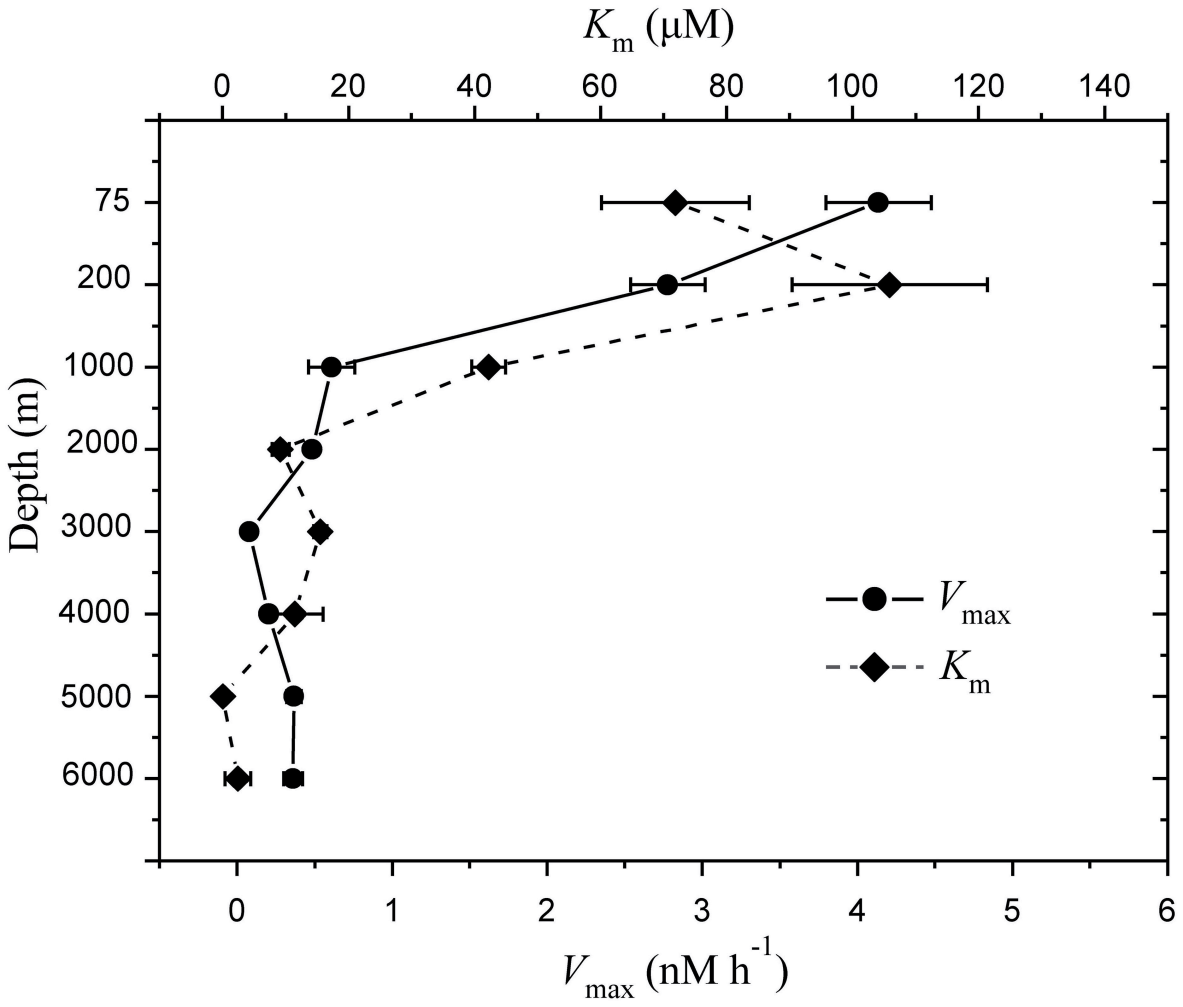
Depth profile of extracellular proteolytic enzyme activities in the water column of the New Britain Trench, showing the mean maximum rates of hydrolysis *V*_max_ (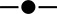) and the half-saturation constants, *K*_m_ (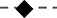).

The depth profiles of aminopeptidase activity were found distinctly different between the surface water and deep water in the NBT (Figure 5). The *V*_max_-values were much higher in the surface water with a maximum value of 4.14 nM h^−1^ at 75 m and then decreased continuously with depth, with the lowest value of 0.078 nM h^−1^ at 3,000 m. The average *V*_max_ were ~10-fold higher in the surface waters (75 and 200 m) than that in the deep waters (Mann–Whitney test; *P* < 0.05). Similarly, the half saturation constant (*K*_m_) also showed significant decrease with depth (Mann–Whitney test; *P* < 0.05). Interestingly, the *K*_m_ and *V*_max_ profiles exhibited a mirrored relationship (Figure 5).

## Discussion

Hydrolytic ECEs of marine bacteria are of fundamental importance for microbial processing of polymeric organic matter in the ocean (Somville and Billen, 1983; Ammerman and Azam, 1985; Arnosti, 2014). Although extracellular proteolytic activity in the surface ocean and the mesopelagic zone has been fairly well-studied (Christian and Karl, 1995; Hoppe and Ullrich, 1999; Obayashi and Suzuki, 2005; Baltar et al., 2009), there have been no investigations of cultivable enzyme-producing bacteria and their ECEs in the trench environment. Our study provides new insights on the diversity of cultivable bacteria and the stratified bacteria enzyme profiles in the abyssal water column of the New Britain Trench.

### Cultivable bacteria community composition differs among different pelagic zones

In this study, 10^3^-10^5^ bacterial cells ml^−1^ were counted from the abyssopelagic to surface seawater in the NBT, and 10^2^-10^4^ cells ml^−1^ could be cultivated from the seawater of different depths. The average cultivable rate (cultivable population vs. total cell population) of the bacteria was 8%, which is much higher than the commonly reported 1% in the literature (Jensen et al., 1996; Bernard et al., 2000).

The diversity and community structure of the cultivable bacteria varied with depth in the NBT (Figure 2). The 75 bacterial isolates belong to 10 genera in two phyla, Proteobacteria and Actinobacteria, with the former dominated in all depths except at 2000 m. *Gammaproteobacteria* is widely distributed in the marine water column and sediment, represented mainly by *Pseudoalteromonas* as the dominant genus (Zhou et al., 2009; Qin et al., 2011; Li et al., 2015; Degli Esposti and Martinez Romero, 2017). The synthesis of highly active proteolytic enzymes is a widespread characteristic for members of the genus *Pseudoalteromonas* of the class *Gammaproteobacteria* (Ivanova et al., 2004; Mikhailov et al., 2006; Tropeano et al., 2012). Similarly, in our study, 81.3% of the cultivable bacteria were detected to produce ECEs, and the protease-producing genera were dominated by *Pseudoalteromonas* (46 out of 53) over the other groups of bacteria (Figure 3). This result corroborates the recent finding that protease-producing bacteria in sediment porewater of the South Pacific Gyre distributed primarily in *Pseudoalteromonas* genus of the *Gammaproteobacteria* (Zhang et al., 2016). Moreover, previous studies found that *Gammaproteobacteria* was the predominant cultivable protease-producing bacteria in marine sediments (Zhou et al., 2009; Zhang et al., 2015; Li et al., 2017). Extracellular proteolytic activity seems to be well-represented among members of *Pseudoalteromonas* in the marine environment (Holmström and Kjelleberg, 1999; Vazquez et al., 2004; Xiong et al., 2007). Our results indicate the existence of sizable populations of protease-producing *Gammaproteobacteria* in the water column of the New Britain Trench and likely in all different marine environments.

### Depth-resolved variations of bacterial ECEs

It can be seen that 47 of the 61 enzyme-producing bacteria could degrade at least two different substrates (Table S1), suggesting that marine microorganisms often produce ecologically relevant enzymes (Becker et al., 2017), depending on the availability of the growth substrates. It follows that the existence of the multiple enzyme-producing microorganisms allows them to take advantage of a broader spectrum of available substrates in the water column for carbon and nutrients, and this also provides a small glimpse into the adaption of the bacteria to the trench environment. Bacteria produce ECEs in response to available substrates. In general, organic matter in the surface water is relatively fresh and is probably consisted of all different constituents (e.g., carbohydrates, lipids, and proteins; Hoefs et al., 1997; Fabiano and Pusceddu, 1998). Thus, all major types of ECEs could be expressed by the resident bacteria in the surface water. In our study, we found substantial changes in enzymatic capabilities of the cultivable bacteria in both the surface and deep waters (Figure 1), with a more diverse set of enzymes (amylase, esterase, and proteases-degrading casein and gelatin) in the surface water. In the meso- and bathypelagic waters, however, amylase was not detected and esterase increased concurrently, suggesting that carbohydrates were probably not a major component of the DOM in the deep waters of the NBT. As proteases were the dominant enzymes secreted by most of the cultivable enzyme-producing bacteria in this study, we determined the diversity of the extracellular proteases of the cultivable bacteria in the NBT water column. We found that the bacterial extracellular proteases show a clear depth trend, with mostly serine proteases in the surface water and metalloproteases in the deep water. These results agree with findings in previous studies (Arnosti et al., 2011; Steen et al., 2012; Tropeano et al., 2012; D'Ambrosio et al., 2014; Hoarfrost and Arnosti, 2017) and have important implications for microbial physiology and biogeochemical cycle in the ocean. Pantoja and Lee (1999) found that rates of peptide hydrolysis were greatly affected by the size and structure of the substrates; for example, peptides with more than two amino acids were hydrolyzed faster than dipeptides in natural seawater. In our study, protease-producing isolates showed preference for hydrolyzing gelatin compared with casein in the water column of the NBT, this could be accounted for by the bacterial adaptation to marine biochemical cycle, in which organic nitrogen is mainly consisted of high-molecular-weight-combined amino acids (Keil and Kirchman, 1994). These results demonstrate that substrate bioavailability and biodegradability depend on not only the chemical and structural characteristics of the substrates, but also on the enzymatic capabilities of the microbial community.

### Stratified proteolytic activities, substrate affinity, and utlization

Within the water column of the NBT we found large variations in aminopeptidase activity with depth. Both *V*_max_ and *K*_m_-values in the surface waters were much higher than those in the deeper waters (Mann–Whitney test; *P* < 0.05), indicating lower rates of hydrolysis and higher substrate affinity in the deep. Similar findings were reported for proteolytic activities at the surface (< 100 m) and deep waters (500–2000 m) of the Santa Monica Basin (Rosso and Azam, 1987) and the northwestern Mediterranean Sea (Tamburini et al., 2002). In relatively oligotrophic environments such as the deeper water of the trenches where organic matter is supposed to be low in content and more refractory in nature, high substrate affinity would enable heterotrophic bacteria to process the maximum amount of the organic matter available. The significant correlation (*R* = 0.978, *P* < 0.0001) between the maximum potential proteolytic activity and bacteria numbers in the NBT is not unexpected, as previous studies have shown that bacterial extracellular enzymes remain in close association with the cells (Martinez and Azam, 1993; Davey et al., 2001; Celussi et al., 2009), suggesting that the proteolytic activity in the NBT can be attributed to the resident heterotrophic bacteria (e.g., Rosso and Azam, 1987).

However, cell-specific (*V*_max_/bacterial counts) proteolytic activities (0.037–0.082 pmol cell^−1^h^−1^) showed greater variations than that observed in other areas (e.g., the Mediterranean Sea; Tamburini et al., 2009). These authors found that cell-specific activity was typically higher at depth than in surface seawater in the Mediterranean Sea. In this study we found that the cell-specific proteolytic activities (0.037–0.082 pmol cell^−1^h^−1^) were highest in the lower part of the abyssopelagic water (5000–6000 m), and lowest (0.014–0.015 pmol cell^−1^h^−1^) at 3000–4000 m (Table 1). This finding may be explained by the reduced quality and quantity of DOM and its importance for microbial metabolism and carbon cycling in the deep ocean. The decreasing availability of labile organic matter with depth leads to an increase in cell-specific extracellular enzymatic expression and concomitantly, to a reduction in the prokaryotic growth yield. The increase in the cell-specific extracellular enzymatic activity with depth further indicates an adaption of the extracellular prokaryotic enzymes to the refractory nature of the organic matter at depth (Baltar et al., 2009).

## Conclusions

It has been proposed that particle-attached and free-living microorganisms in the water column play an important role in oceanic carbon cycle. Here, we present vertical profiles of cultivable enzyme-producing bacteria and bacterial extracellular enzymes from the surface to the abyssopelagic zone in the New Britain Trench. It is clear that the both the cultivable bacteria and ECEs exhibit stratified distributions, showing varying abundance of cosmopolitan taxa and the presence of unique clades. These lineages may be responsible for the stratification of the ECEs in the water column. In addition, the *V*_max_ and *K*_m_-values of total proteolytic enzyme exhibited highest values in the surface water and lowest in the abyssopelagic zone, suggesting reduced rates of hydrolysis and increased substrate affinity in the deeper water. Our findings have important implications for understanding the dynamics of the microbially-driven ocean carbon cycle, particularly on the connection among microbial community composition, enzyme expression, and capabilities in the context of the trench environment (e.g., the chemistry of organic matter) (Arnosti, 2014; Fang et al., 2015). The variations with depth in the diversity of cultivable bacteria, their ECEs and activities probably reflect the reduced quality and quantity of DOM in the deep ocean and concomitant microbial adaptation to the changing deep ocean environmental conditions. Therefore, microbial community structure and enzyme production controls the overall patterns of organic matter flux and carbon cycle in the oceans.

It is well-known that culture-dependent approaches have certain biases (e.g., Giovannoni and Stingl, 2007), thus, it is possible that ECEs of the uncultivable microbes may be different from those of the cultivable ones. Hence, it is beneficial to combine the traditional culturing approaches with metagenomic and metatranscriptomic technologies to explore functional microorganisms and their enzymatic activities in the ocean. Nevertheless, this study represents the first to study both the cultivable bacteria and the activity of their ECEs in the abyssal water column, providing new insight into the diversity and activity of cultivable bacteria and their ECEs in the trench environment.

## Author contributions

JF and Y-ZZ: designed the research; QL: collected the samples and conducted the experiments; QL: wrote the manuscript with significant input from JF. JL and LZ: participated in part of the experiment; X-LC and B-BX: performed the inhibition test of protease. All authors contributed to the interpretation of the data.

### Conflict of interest statement

The authors declare that the research was conducted in the absence of any commercial or financial relationships that could be construed as a potential conflict of interest.
