# Supplementary material for: Depth-Resolved Variations of Cultivable Bacteria and Their Extracellular Enzymes in the Water Column of the New Britain Trench
Source: Front Microbiol. 2018 Feb 6;9:135. doi: 10.3389/fmicb.2018.00135 (PMC5808245; doi:10.3389/fmicb.2018.00135)
Supplement: Supplementary file 1 [file DataSheet1.docx]

Supplementary Material

**Depth-Resolved Variations of Cultivable Bacteria and their Extracellular Enzymes in the Water Column of the New Britain Trench**

**Qianfeng Liu, Jiasong Fang^*^, Jiangtao Li, Li Zhang, Bin-Bin Xie^*^, Xiu-Lan Chen, Yu-Zhong Zhang**

*** Correspondence:** Jiasong Fang: [jsfang@shou.edu.cn](mailto:jsfang@shou.edu.cn); Bin-Bin Xie: [xbb@sdu.edu.cn](mailto:xbb@sdu.edu.cn)

# Supplementary Figures and Tables

## Supplementary Figure

**Supplementary Figure 1.** The neighbor-joining phylogenetic tree of the strains in Branch 1 of Figure 3 based on the 16S rRNA gene sequences.

**Supplementary Figure 2.** The neighbor-joining phylogenetic tree of the strains in Branch 2 of Figure 3 based on the 16S rRNA gene sequences.

**Supplementary Figure 3.** Epifluorescence photomicrographs of DAPI-stained microbial communities in sea water sample from 75 m (A) and 3000 m (B). Scale bar = 5 μm.

## Supplementary Table

| **Supplementary Table 1.** H/C ratios of strains producing extracellular enzymes and diversity analysis of the extracellular proteases | | | | | | | | |
| --- | --- | --- | --- | --- | --- | --- | --- | --- |
| Depths | Strains | H/C ratio^a^ | | | | Inhabiton ratio (%)^b^ | |  |
|  |  | Casein | Gelatin | Starch | Triacetin | PMSF (1 mM) | OP (1 mM) |  |
| 75m | NBTE-P21 | 4.3 | 3.1 |  |  | 96.4 | 11.6 |  |
|  | NBTE-P3 | 3.2 | 6.8 |  |  | 96.2 | 21.5 |  |
|  | NBTE-P4 | 3 | 4.8 |  |  | 98.6 | 14.4 |  |
|  | NBTE-P19 | 2.8 | 7.7 |  |  | 96.7 | 7.8 |  |
|  | NBTE-P5 | 2.5 | 5.6 |  |  | 98.9 | 6.9 |  |
|  | NBTE-P16 | 2.4 | 10.4 |  |  | 80.2 | 37.8 |  |
|  | NBTE-P1 | 2.3 | 4 |  |  | 86.9 | 14.6 |  |
|  | NBTE-P28 | 1.5 | 7.4 |  |  | 97.4 | 5.1 |  |
|  | NBTE-P26 | 1.4 | 6 |  |  | 97.9 | 22.9 |  |
|  | NBTE-P18 |  | 9.1 | 4 | 2.3 |  |  |  |
|  | NBTE-P22 |  | 2.7 | 3.1 | 3.5 |  |  |  |
| 200m | NBTE-Q3 | 2.7 | 7.7 |  |  | 99.2 | 10.7 |  |
|  | NBTE-Q8 | 2.6 | 7.6 |  |  | 99.3 | 23.6 |  |
|  | NBTE-Q12 | 2.6 | 6.5 |  |  | 96.5 | 9.9 |  |
|  | NBTE-Q1 | 2.6 | 7.6 |  |  | 99.8 | 4.5 |  |
|  | NBTE-Q25 | 2.3 | 4.2 |  |  | 99.2 | -0.5 |  |
|  | NBTE-Q4 | 2.2 | 5.2 |  |  | 86.2 | -5.9 |  |
|  | NBTE-Q17 | 2 | 8 |  |  | 95.1 | 2.7 |  |
|  | NBTE-Q5 | 1.7 | 6.3 |  |  | 99.8 | -12.9 |  |
|  | NBTE-Q16 |  | 6.6 | 3.1 | 4 |  |  |  |
|  | NBTE-Q23 |  | 2.3 |  |  |  |  |  |
|  | NBTE-Q24 |  | 1.3 |  |  |  |  |  |
| 1000m | NBTE-R3 | 1.5 | 2.9 |  |  | 79.8 | 36.5 |  |
|  | NBTE-R10 | 1.4 | 2.4 |  |  | 86.1 | 38.0 |  |
|  | NBTE-R8 | 1.4 | 2.5 |  |  | 85.5 | 12.4 |  |
|  | NBTE-R7 | 1.4 | 1.6 |  |  | 63.4 | 35.6 |  |
|  | NBTE-R1 | 1.3 | 1.4 |  |  | 81.6 | 39.5 |  |
|  | NBTE-R2 | 1.2 | 2.4 |  |  | 77.1 | 31.7 |  |
|  | NBTE-R19 |  | 7.7 |  | 5.8 |  |  |  |
|  | NBTE-R14 |  | 1.9 |  |  |  |  |  |
| 2000m | NBTE-S1 | 3.8 | 9.3 |  |  | 97.7 | 14.1 |  |
|  | NBTE-S3 | 3.8 | 5.7 |  |  | 99.6 | 15.9 |  |
|  | NBTE-S25 |  |  |  | 6.3 |  |  |  |
| 3000m | NBTE-T1 | 2.5 | 7.3 |  |  | 95.4 | 7.4 |  |
|  | NBTE-T4 | 1.7 | 9.9 |  |  | 98.7 | 5.2 |  |
|  | NBTE-T3 | 1.5 | 6.5 |  |  | 95.3 | 7.8 |  |
|  | NBTE-T14 |  | 2.6 |  |  |  |  |  |
|  | NBTE-T12 |  | 2.4 |  |  |  |  |  |
| 4000m | NBTE-W15 | 3.1 | 4.3 |  |  | 89.9 | -2.2 |  |
|  | NBTE-W3 | 2.7 | 5.6 |  |  |  |  |  |
|  | NBTE-W1 | 2 | 4 |  |  |  |  |  |
|  | NBTE-W4 | 1.8 | 7.9 |  |  |  |  |  |
|  | NBTE-W13 |  |  |  | 9.2 |  |  |  |
|  | NBTE-W14 |  |  |  | 7.3 |  |  |  |
|  | NBTE-W20 |  |  |  | 6.7 |  |  |  |
| 5000m | NBTE-X2 | 3.2 | 7.5 |  |  | 96.2 | 10.5 |  |
|  | NBTE-X1 | 2.8 | 6.3 |  |  | 80.7 | -0.7 |  |
|  | NBTE-X19 |  |  |  | 8.1 |  |  |  |
|  | NBTE-X12 |  |  |  | 7.4 |  |  |  |
|  | NBTE-X22 |  |  |  | 5.5 |  |  |  |
| 6000m | NBTE-Y1 | 5.6 | 3.7 |  |  | 48.6 | 45.7 |  |
|  | NBTE-Y3 | 4.9 | 3.9 |  |  | 47.2 | 6.4 |  |
|  | NBTE-Y19 | 4.7 | 4.5 |  |  | 43.4 | 50.4 |  |
|  | NBTE-Y2 | 4.7 | 2.6 |  |  | 46.9 | 42.6 |  |
|  | NBTE-Y15 | 4.2 | 3.3 |  |  | 44.5 | 37.3 |  |
|  | NBTE-Y6 | 3.9 | 4.3 |  |  | 42.2 | 16.8 |  |
|  | NBTE-Y7 | 3.9 | 4.7 |  |  | 43.4 | 15.9 |  |
|  | NBTE-Y5 | 3.7 | 5.5 |  |  | 38.0 | 38.8 |  |
|  | NBTE-Y13 | 2.6 | 4.2 | 1.4 |  | 43.9 | 76.3 |  |
|  | NBTE-Y18 |  |  |  | 6.5 |  |  |  |

PMSF phenylmethylsulfonyl fluoride, OP 1,10-phenanthroline

^a^ H/C ratio is the ratio of the hydrolytic zone diameter to the colony diameter of a colony on the plate

^b^ The activity of a sample without any inhibitor was taken as control (100%). The inhibition ratio was taken as the result of control activity minus the relative activity of a sample with an inhibitor
